# Comparison of Students’ and Teachers’ Opinions Toward Digital Citizenship Education

**DOI:** 10.3389/fpsyg.2021.752059

**Published:** 2021-11-08

**Authors:** Hasan Tangül, Emrah Soykan

**Affiliations:** ^1^Department of Computer Education and Educational Technologies, Faculty of Education, Kyrenia University, Kyrenia, Cyprus; ^2^Department of Computer Education and Educational Technologies, Faculty of Education, Near East University, Nicosia, Cyprus

**Keywords:** communication in COVID-19, digital citizenship, digital citizenship education, digitalization, digital transformation

## Abstract

The impact of the COVID-19 pandemic has led to the temporary interruption of educational activities in the classroom. Digitalization of the classrooms emerged as a need following that process. The objective of this study is to compare the digital citizenship levels of teacher candidates studying in the last year at the departments of classroom teaching and primary school classroom teachers and to reveal their needs. A total of 38 primary school classroom teachers and 27 classroom teacher candidates in the last year of teaching programs in North Cyprus participated in the research. This descriptive study was designed as a case study, which is a qualitative research approach. The data of the study were gathered within the frame of a grounded theory coding process and were analyzed through descriptive analysis, content analysis and the constant comparison technique. The digital citizenship levels of the classroom teachers and teacher candidates were analyzed according to the sub-dimensions of digital citizenship. As a result of the research, it was found that the digital citizenship sub-dimension scores of the classroom teachers were higher than the teacher candidates and that the teacher candidates needed digital citizenship education.

## Introduction

As a result of the continuingly developing modern technologies, distance has become unimportant, which has led to a fundamental transformation of human life (Davies, 2011). This change has triggered human curiosity toward technology and the Internet and the levels of interest and addition to the Internet are continually increasing. Furthermore, it is now virtually compulsory to use the Internet in modern society (Akkoyunlu and Yılmaz, 2005). New generations are exhibiting significant differences to previous generations in terms of technology use. However, in the last decade alone, the regular access and use of digital technology have increased in all age groups (Rideout, 2017).

Parallel to the increase in use of the Internet, the development of mobile devices, information communication technologies and cloud technologies have also had a considerable impact. In particular, desktop computers, laptops, smart phones, and tablets, and other mobile devices allow humans to integrate the digital world into their daily lives and they can now easily contact their friends *via* online media. Also, people have started to use mobile devices in schools or other environments for educational purposes as the technology can be used anywhere at any time. The use of digital devices during the ongoing COVID-19 pandemic that has impacted the world has also become compulsory and many people around the world are using them to meet their needs, such as for shopping, communication, and education (Akcıl and Bastas, 2021). It is observed that individuals are taking measures such as isolating themselves or protecting social distance (Ranchordas, 2020). In the studies conducted by Yolal and Kozak (2008), it was found that current users started to meet their needs through electronic environments and this situation highlighted the concept of digital life. Another factor is that individuals are able to transfer their knowledge to each other by using online media, use all digital applications provided by governments and access news from around the world immediately. In other words, people have begun to meet their needs through the digital media such as e-shopping, e-government, e-books, e-libraries, e-Pulse (e-Nabiz), e-banking, etc.

As a result of the impact of technological developments on people’s daily routines, the number of technology users continues to increase. Through these developments, technology has become fundamentally integrated into people’s lives and has added a new dimension to human relations and communication, such that a new societal concept has emerged. This concept is called the network society and the members of this society are called digital citizens (Castells, 2000). The digital citizen uses their own communication possibilities and their own knowledge while engaging in critical thinking and understanding the ethical causes of the behaviors they face and exhibit in the digital media. They are citizens who can use technology without harming people who benefit from the communication right on the digital media as well as showing the right attitude while sharing anything on digital media which paves the way for its being a model for the people toward those attitudes (Çubukcu and Bayzan, 2013). Furthermore, in the study by Çubukcu and Bayzan (2013), digital citizenship was defined in general terms as the behavioral norms that occur in the integration of the rights and responsibilities related to technology use. In summary, digital citizenship can be defined as the adoption of appropriate attitudes and behaviors of individuals while using technology by assuming responsibility for their actions (Mossberger et al., 2007).

In a study by Ribble (2015), all the states of the concept of digital citizenship were analyzed, which he then separated into nine dimensions: “Digital Access, Digital Trade, Digital Communication, Digital Literacy, Digital Ethics, Digital Law, Digital Rights and Responsibilities, Digital Health, and Digital Security.” As technology has become essential in our daily lives, it is important that it is used safely and effectively. Digital citizenship refers to the ability to use technology correctly and appropriately rather than merely using it. As technology gains more importance, the digital citizenship concept will also become more important. An examination of the studies conducted in this field reveals that most of them focused on citizens and their privacy, security, accessibility by everyone through technology as well as their communication possibilities and their health and ethical dimensions (Çubukcu and Bayzan, 2013; Kaya and Kaya, 2014). Netwong (2013), conducted a study in order to reveal the relationship between digital citizenship and learning success and also to increase the learning success and digital citizenship in the field of Information Technologies for university students through e-learning. The study group in the research comprised a total of 49 students. The tools used to collect the data included a questionnaire about digital citizenship and the e-learning success test of Suan Dusit Rajabhat University. As a result of the e-learning, the digital citizenship development increased by 15.85%, and the learning success development increased by 23.37%, thus demonstrating that there is a strong correlation between digital citizenship and learning success. Jones and Mitchell (2016), developed a digital citizenship scale for the online civil participation dimensions as well as for respectful online behavior. In their study, they emphasized that there is growing interest toward the improvement of digital citizenship through education. The results showed that younger people exhibited less respectful online behavior and also in terms of the civil participation dimension and respectful behavior, females had higher scores than males. Choi et al. (2017), conducted a study aimed at creating an extensive digital citizenship scale that relates and includes the sub dimensions of digital citizenship which young people could use to measure their participation skills for the Internet based community, their Internet levels and perceptions. In order to determine the sub dimensions of digital citizenship and also to evaluate its reliability and validity, exploratory factor analysis, confirmatory factor analysis, and relational analysis were all used. A total of 72 graduate and 436 undergraduate students at a large western university participated in the research and it was found that the digital citizenship scale measured the students’ skills, perceptions and participation level for the Internet based community in different dimensions. The research emphasized that the scale was effective in terms of informing students, educating them, make them effective digital citizens and providing the necessary conditions for raising the digital citizenship to a higher level.

An examination of the studies included in the literature review shows that all the studies we have analyzed have used scales in order to reveal levels of digital citizenship. Additionally, the researches were generally conducted with students. According to the results of the researches, when training was provided to the participants, they were more informed about digital media and they used this knowledge to achieve their educational aims and these results contributed to the future studies to be used in terms of multiple scales and resources. As people encounter digital media frequently and such forms of media eliminate all the limits related to communication and exposure, the formation of the digital community can be regarded as a very important step. In particular, teacher candidates are faced with digital generations who are exposed to the rapidly changing technologies and exposures, which may cause the candidate teachers to experience problems with regard to technological proficiency and awareness. The frequent use of those media brings together the need to evaluate the program contents as well as need to consider the current state to ensure that students develop a suitable level of digital citizenship awareness. Therefore, a needs analysis for primary classroom teachers and candidate teachers in the last year of their studies should be performed to determine their digital citizenship levels and the development of “Digital Citizenship Education” to meet those needs is very important. Accordingly, the aim of this study is to determine the digital citizenship levels of primary classroom teachers and the candidate teachers in their last year at the university as well as to compare them and reveal their needs. Based on this aim, a needs analysis was conducted.

## Method

### Research Model

A case study model was used, which is a qualitative research pattern. In case studies, an event, situation or a program is analyzed and evaluated according to different dimensions (Yin, 2017). The extensively and systematically obtained data for the current situation/s in this process are holistically analyzed and the current situation is then assessed and described in great detail (Johnson and Onwuegbuzie, 2004; Yıldırım and Şimşek, 2008; Patton, 2014).

### Participants

This study was conducted with a total of 65 individuals, comprising 38 primary classroom teachers and 27 candidate teachers studying in the last year at the classroom teaching department during the 2020–2021 fall semester. The participating teachers and candidate teachers were chosen according to the targeted random sampling method.

The aimed random sampling is the assorting the systematically and randomly chosen case samplings according to the objective of the research (Marshall and Rossman, 2014). Moreover, the reliability of the obtained information is considered to be higher when using this method (Creswell, 2016).

### Data Gathering Tool

In order to gather the qualitative data of this research, a literature review was done on the topic and then a “Digital Citizenship Interview Form” was prepared, which included open-ended questions that were developed based on the information acquired from the literature review. This form contained questions related to the nine sub dimensions of digital citizenship and an initial draft was prepared. After that, the draft was presented to five academicians from different universities who were experts in different fields to obtain their feedback. The questions were prepared with the help of the feedback of these experts and they were reshaped according to the paradigm of language and expression and of the qualitative research, resulting in the final version of the form. To determine how long it would take to complete the interviews as well as to evaluate the comprehensibility and answerability of the questions, it was decided that a pilot implementation would be made. Therefore, before sharing the interview form with all the participants, one teacher and two candidate teachers tested the form during the pilot implementation stage to assess the questions’ answerability and time saving features. Subsequently, the questionnaire was sent to participants online by using Google Forms in order to gather the data and feedback from them.

### Data Analysis

The qualitative data of the research were written on computer media and data texts were taken. After this, all the texts were uploaded to the QDA Miner Lite program. The raw data were analyzed by the researchers very carefully before being coded. During the data coding, open, axial, and selective coding techniques were used so as to form a unit for one-sentence-data analysis or word groups or words according to the line-by-line analysis. All coding’s were structured according to the free coding using the qualitative data analysis functionality QDA Miner Lite. As there was no previous code list for the research problem the grounded theory coding process was applied. During the interpretation of open-ended questions; inductive descriptive analysis, content analysis and constant comparison techniques were used. During the content analysis, free coding focused contexts were considered and the created codes were combined under a common category. In the last phase, the cohesion of the themes obtained from the data was ensured and the interpretation process was completed.

## Findings

As illustrated in Table 1, in terms of the technological environment in the school variable, 21 of the classroom teachers (21.6%) expressed that they mostly used computers. However, the candidate teachers used the computers less (13.4%) and preferred using projectors (40.3%). On the other hand, according to the answers for the use of technology outside the school environment variable shows that both classroom teachers (27.8%) and candidate teachers (38.8%) mostly used smartphones. A large proportion of the teachers (81.6%) and the candidate teachers (50%) mentioned that they had not experienced any unethical behavior on digital media.

**TABLE 1:** The distribution of classroom teachers and candidate classroom teachers according to the sub-dimension of digital access and digital ethics.

**Sub dimension**	**Variable**	**Themes**	**Teacher**		**Candidate teacher**	
			**Frequency(**f**)**	**Percent(%)**	**Frequency(**f**)**	**Percent(%)**
Digital access	Technological environment in school	Projection	14	14.4	27	40.3
		Smart board	2	2.1	0	0.0
		Mobile device	9	9.2	0	0.0
	Technological environment outside the school	Smart phone	27	27.8	26	38.8
		Computer	15	15.5	1	1.5
		Tablet	3	3.1	2	3.0

Digital ethics	Unethical behavior	Stealing of social media	3	7.8	0	0.0
		Swearing	2	5.3	5	25.0
		Being disrupted	2	5.3	0	0.0
		Not any	31	81.6	10	50.0

According to Table 2, analysis of the digital security sub dimension indicates that the precautions that the teachers and candidate teachers take vary; most of the teachers prefer “not to share personal and private information” in order to maintain a more secure digital environment, whereas the candidate teachers prefer not to enter to the sites that they do not believe are secure. Some of the teachers (23.3%) gave the answer “forming a group” whereas the remaining teachers said they did not have any information.

**TABLE 2:** The distribution of classroom teachers and candidate teachers according to the sub dimension of digital security and digital rights and responsibilities.

**Sub dimension**	**Variable**	**Themes**	**Teacher**		**Candidate teacher**	
			**Frequency(**f**)**	**Percent(%)**	**Frequency(**f**)**	**Percent(%)**
Digital security	Precautions	Not sharing personal and private information	13	28.9	1	3.2
		Not dealing with unfamiliar people	6	13.3	4	12.9
		Not entering every site	10	22.2	10	32.3
		Putting password	4	8.9	5	16.1

Digital rights and	Fundamental rights	Giving opinion	8	18.6	9	36.0
responsibilities		Forming a group	10	23.3	5	20.0
		I have no information	10	23.3	3	12.0

As illustrated in Table 3, most of the teachers said that they use all digital communication tools (39.7%) according to the answers, while Instagram and Short Message Service (SMS) are used the least (3.2%). The classroom teachers (84.2%) are well informed about the sub dimension of digital law and they were aware of that these were negative should not be used. The candidate teachers mostly benefit from the Internet for reading newspapers (17.0%). On the contrary, six of the teachers said that they prefer not to use the Internet by mentioning that they wanted to benefit from written resources such as newspapers, journals, books, etc.

**TABLE 3:** The distribution of classroom teachers and the candidate classroom teachers according to the sub dimension of digital communication, digital law, and digital literacy.

**Sub dimension**	**Variable**	**Themes**	**Teacher**		**Candidate teacher**	
			**Frequency(**f**)**	**Percent(%)**	**Frequency(**f**)**	**Percent(%)**
Digital communication	Digital communication	WhatsApp	12	19.0	11	33.3
		Viber	5	7.9	0	0.0
		Messenger	8	12.7	1	3.0
		E-mail	6	9.5	5	15.2
		All of them	25	39.7	11	33.3

Digital law	Prohibited broadcasts	I use positively	4	10.5	23	85.2
		Negative/should be banned	32	84.2	4	14.8
		I have no information about the topic	2	5.3	0	0.0

Digital literacy	Benefiting from the internet	Newspaper	15	17.0	4	8.5
		All	14	15.9	7	14.9
		Google	22	25.0	11	23.4

The responses of the teachers and the candidate teachers regarding the negative effects on health are revealed in Table 4. According to the teachers, the digital environment had both physical and psychological negative effects (52.8%), whereas according to the candidate teachers, the negative effects are more psychological (37.5%). The majority of the classroom teachers thought that online shopping is insecure (21.7%), so they do not shop online.

**TABLE 4:** The distribution of classroom teachers and candidate classroom teachers according to the sub dimension of digital health and digital trade.

**Sub dimension**	**Variable**	**Themes**	**Teacher**		**Candidate teacher**	
			**Frequency(**f**)**	**Percent(%)**	**Frequency(**f**)**	**Percent(%)**
Digital health	Negative effects to health	Physical	10	27.8	7	29.2
		Both physical both psychological	19	52.8	5	20.8
		I have no information about the topic	4	11.1	2	8.3

Digital trade	Online shopping	Time saving	6	7.2	18	39.1
		More reasonable price	12	14.5	1	2.2
		Not using due to insecurity	18	21.7	3	6.5
	
	Internet banking	Time saving	22	26.5	11	23.9
		Not using due to insecurity	6	7.2	1	2.2

## Discussion

As a result of the sub dimension of the digital access of the digital citizenship, both the classroom teachers and the candidate classroom teachers used projectors and computers inside the school, whereas outside the school, they mostly used smart phones by actively benefiting from the digital technology This result shows adjustment with the results of the study conducted by Vural and Kurt (2018) and it is concluded that individuals from all areas of society are able to access digital equipment-tools and the applications in their daily lives, especially through mobile devices.

In terms of the sub dimension of digital ethics, a large majority of both the classroom teachers and the candidate classroom teachers mentioned that they had not faced any unethical behavior in the digital environment. Three classroom teachers said that their social media accounts had been hacked, two classroom teachers and five candidate classroom teachers said they had experienced swearing in the digital environment and two candidate classroom teachers also mentioned that they had encountered fake accounts on the digital environment. Regarding these results, both the classroom teachers and the candidate classroom teachers stated that they had insufficient knowledge about digital ethics and needed to receive seminar style trainings. There has been a serious increase in the rate of cybercrimes and there have not been sufficient informative studies on this subject, indicating that it requires more attention Çolak et al. (2011). These opinions support the view mentioned above. In his doctoral thesis, Sari (2019) conducted a seminar on the behaviors of teachers in terms of the ethical use of digital technologies and found that this seminar had a positive effect on the teachers. After this seminar, the teachers began to behave more sensitively in terms of obeying the ethical values on the observed digital environment.

Within the frame of the sub dimension of digital security; both the classroom teachers and the candidate classroom teachers attached importance to digital security. The participants stated that they did not share private information on the digital environment, did not deal with unfamiliar people, they used antivirus programs, used passwords and changed them regularly. Similarly, the results of the study conducted by Symantec (2010) were similar to ours and it was found that children were aware of the rules about secure browsing in the online environment, but they could not keep pace with the rapid changes in the online environment. Our results are not in agreement with those of Takavarasha et al. (2018), who emphasized that individuals did not consider the digital security factor.

During the interview with the classroom teachers and the candidate classroom teachers, in terms of digital rights and responsibilities, they stated that they were citizens who actively join to express their opinions effectively, comment, form groups and who participate in discussions. However, 10 classroom teachers and 3 candidate classroom teachers said that they did not have any information about the topic as well as being insufficient about it. According to the literature Tan and Merey (2021) students were aware of the right to freedom of expression, obtaining information and communication rights and they also knew that rights and the responsibilities that humans have in their social lives also exist in the digital environment.

In terms of the sub dimension of digital law, the classroom teachers were more aware of the digital laws than the candidate classroom teachers. The candidate classroom teachers did not have information about the digital laws, therefore they downloaded videos, films, music, games, etc., without permission in terms of plagiarism and they did not make references to the information they gathered from the Internet for their homework. This result of the research has in agreement with the results of Beder (2015). In his study, it was found that secondary school students did not use the Internet securely, they shared posts that would cause problems and they plagiarized online materials. On the other hand, Aslan and Çakmak (2018) analyzed this dimension as digital right and responsibility dimension and at this point, they found out that the participants behaved consciously but sometimes in deliberately sometimes they could not obey the plagiarism rules. The participants stated that they did not follow the rules mostly in relation to downloading films and music.

An examination of the digital literacy sub dimension revealed that the classroom teachers had used their digital literacy to access online news websites, up-to-date journals or books and also preparing lessons on the digital environment. According to Sarsar and Engin (2015), a good digital literate teacher is a model for his or her students. As the teachers are connected to the generations to raise in their own effect area, it is important for this generation to grow up with the most meaningful and up-to-date knowledge. With the findings that Onursoy (2018) obtained, within the use of the technology by the youngsters their digital literacy levels are parallel to this use but their literacy is lower.

Analysis of the digital trade sub dimension shows that both the classroom teachers and the candidate classroom teachers engaged in online shopping. As part of the digital trade the Internet banking theme was used by the classroom teachers especially due to the fact that it saves time and they preferred this type of banking instead of going directly to the bank. In their study Kaya and Kaya (2014) stated that candidate teachers did shopping in the online environment. Another study Aslan and Çakmak (2018) stated that the candidate teachers made e-payments *via* credit card to pay Public Personnel Selection Exam (KPSS) or Student Selection and Placement Exam (ÖSYM) exam application fees, but other than this, they tried not to do online shopping *via* unsecure sites. Furthermore, the candidate teachers mostly perceived online trade to be banking and online bank account follow-up, which is similar to the results of our study.

## Data Availability Statement

The raw data supporting the conclusions of this article will be made available by the authors, without undue reservation.

## Ethics Statement

The authors assert that all procedures contributing to this work comply with the ethical standards of the relevant national and institutional committees on human experimentation. All participants gave written informed consent in accordance with the Declaration of Helsinki. The study was approved by the Scientific Board of Near East University. The patients/participants provided their written informed consent to participate in this study.

## Author Contributions

HT designed and carried out the study and contributed to the analysis of the results and to the writing of the manuscript. ES designed and carried out the study, collected data, and contributed to the writing of the manuscript. Both authors contributed to the article and approved the submitted version.

## Conflict of Interest

The authors declare that the research was conducted in the absence of any commercial or financial relationships that could be construed as a potential conflict of interest.

## Publisher’s Note

All claims expressed in this article are solely those of the authors and do not necessarily represent those of their affiliated organizations, or those of the publisher, the editors and the reviewers. Any product that may be evaluated in this article, or claim that may be made by its manufacturer, is not guaranteed or endorsed by the publisher.
